# Proton Conduction in Acceptor-Doped BaSnO_3_: The Impact of the Interaction between Ionic Defects and Acceptor Impurities

**DOI:** 10.3390/ma15144795

**Published:** 2022-07-08

**Authors:** Lev Putilov, Vladislav Tsidilkovski

**Affiliations:** Institute of High-Temperature Electrochemistry, 20 Akademicheskaya St., 620990 Ekaterinburg, Russia

**Keywords:** proton conduction, acceptor-doped oxide perovskites, acceptor impurities, trapping, BaSnO_3_

## Abstract

Barium stannate is known as a promising proton-conducting material for clean energy applications. In this work, we elucidate the effect of the interaction of protons and oxygen vacancies with acceptor impurities on proton conduction in acceptor-doped BaSnO_3_. The analysis relies on our theoretical developments in hydration and proton hopping in proton-conducting perovskites. The transport theory, based on the master equation and effective medium approximation, provides the analytical description of hopping conduction considering the effects of disorder and changes in the potential energy landscape for protons caused by acceptor impurities. Using the proposed approach, we establish the dependence of the proton mobility and conductivity on the energies of the acceptor-bound states of ionic defects and external conditions. It is shown that the considered interactions can substantially affect the effective activation energies and prefactors of these transport coefficients. We also demonstrate that the correlation between the ionic radius *r*_A_ of an acceptor impurity and the energies of its interaction with ionic defects leads to a non-monotonic dependence of the proton conductivity on *r*_A_. The obtained results are in reasonable agreement with the experimental data on the bulk conductivity of BaSnO_3_ doped with different acceptors.

## 1. Introduction

Acceptor-doped proton-conducting oxides are garnering significant attention due to their potential use in clean energy applications such as protonic ceramic fuel cells and electrolyzers [1,2,3,4]. Acceptor impurities, required for the hydration and appearance of protonic charge carriers, can substantially affect various properties of proton-conducting oxides. Tuning the properties important for applications by optimal acceptor doping is one of the key issues in the development of these materials. The influence of acceptor impurities on hydration and proton transfer has been extensively studied in both experimental [5,6,7,8,9,10,11] and theoretical [12,13,14,15,16,17,18,19,20,21] works. Abundant evidence has been obtained showing that the interaction between acceptor impurities and ionic defects is of fundamental importance for these phenomena in proton-conducting oxides. The main theoretical results on the influence of such interactions on proton transport were obtained by computer simulations—in particular, by the Monte Carlo method [18,19,20,21]. In our recent study [22], we proposed an analytical theory of proton conduction in acceptor-doped perovskites accounting for the fundamental effects caused by acceptor impurities (disorder, acceptor-bound defect states, changes in the potential energy landscape for proton hopping, percolation effect). This approach, which relies on the master equation for proton hopping and effective medium approximation, allowed us to describe the experimental data on the proton conductivity of BaZr_1–x_R_x_O_3–δ_ [22] (R here and below denotes an acceptor impurity).

In this work, based on the theoretical developments in hydration [8,16] and proton hopping conduction [22], we explore the impact of the interaction between impurities and ionic defects on proton transport in acceptor-doped BaSnO_3_. High proton conductivity and chemical stability make acceptor-doped BaSnO_3_ potentially interesting as a proton-conducting material [7,23,24]. Due to its high electron mobility, n-type BaSnO_3_ is a promising material for electronic applications [25,26,27]. We have recently demonstrated that barium stannate is a good model object to analyze the role of the interaction of protons and oxygen vacancies with acceptor impurities in the hydration of proton-conducting oxides [8]. Taking into account this interaction allowed us to explain the effect of dopant type and concentration on the observed hydration behavior of BaSn_1–x_R_x_O_3–δ_ [8].

Here, we elucidate the effect of the considered interactions on the dependence of the proton mobility *u*_H_ and conductivity σ_H_, as well as their effective activation energies and prefactors, on external conditions for acceptor-doped BaSnO_3_. The relationships between the studied transport characteristics and the trapping energies of ionic defects by acceptor impurities are established. It is shown that the low-temperature behavior of σ_H_ and its effective activation energy $E_{a}^{\sigma }$ is determined by the proton trapping energy, while, at higher temperatures, σ_H_ and $E_{a}^{\sigma }$ depend on the trapping energies of both protons and oxygen vacancies. This, in particular, can alter the order in which the values of σ_H_ and $E_{a}^{\sigma }$ corresponding to different impurities increase as temperature changes. To compare our findings with experiments, we exploit the values of the trapping energies for specific impurities determined by the DFT method [28]. The obtained theoretical results are in reasonable agreement with the experimental data on the bulk proton conductivity of BaSn_1–x_R_x_O_3–δ_ [7,29].

In addition, we analyze the implications of the trapping effect for the dependence of the proton conductivity on the ionic radius *r*_A_ of the acceptor impurity in BaSnO_3_. The calculated dependence σ_H_(*r*_A_) is non-monotonic, in accordance with the experimental observations for BaSn_1–x_R_x_O_3–δ_ and other acceptor-doped perovskites.

## 2. Theory

### 2.1. Hydration

To describe the hydration of barium stannate, we exploit the statistical approach recently proposed to elucidate the effect of acceptor-bound complexes of ionic defects on the hydration and defect thermodynamics of ABO_3_ perovskites [16]. Herein, we consider hydration taking into account two-particle complexes formed by acceptor-bound protons and oxygen vacancies. In this model, there exist two types of states for ionic defects. These states, bound and free, correspond to oxygen sites located in the vicinity of acceptor impurities and away from them, respectively. In our previous studies [8,16], we demonstrated that the implemented approach allows one to correctly describe the hydration of acceptor-doped perovskites BaZrO_3_, BaCeO_3_ and BaSnO_3_.

In the exploited model [16], the concentration of protons *c*_H_ in cubic perovskites AB_1–x_R_x_O_3–δ_ in the case of moderate dopant content can be written as

(1) $$c_{H}=\frac{3}{4}K_{\mathrm{hydr}}p_{H_{2}O}\left(-1+\sqrt{1+\frac{8c_{R}}{3K_{\mathrm{hydr}}p_{H_{2}O}}}\right).$$

Here, *p*_H_2_O_ is the partial pressure of water vapor (in atm); *c*_R_ is the concentration of acceptor impurities (per formula unit); *K*_hydr_ is the equilibrium constant of hydration, given by:

(2) $$K_{\mathrm{hydr}}=exp\left(-\frac{\Delta H_{\mathrm{hydr}}^{0}-T\Delta S_{\mathrm{hydr}}^{0}}{kT}\right)K_{\mathrm{hydr}}^{\mathrm{trap}},$$

where $\Delta H_{\mathrm{hydr}}^{0}$ and $\Delta S_{\mathrm{hydr}}^{0}$ are the enthalpy and entropy of hydration in the absence of trapping; $K_{\mathrm{hydr}}^{\mathrm{trap}}$ is the component of the equilibrium constant associated with trapping. Within the adopted approach, $K_{\mathrm{hydr}}^{\mathrm{trap}}$ can be expressed as [16]

(3) $$K_{\mathrm{hydr}}^{\mathrm{trap}}=\frac{{\left[p_{f}+p_{b}exp\left(\Delta E_{H}/kT\right)\right]}^{2}}{p_{f}+p_{b}exp\left(\Delta E_{V}/kT\right)},$$

where Δ*E*_H_ and Δ*E*_V_ are the trapping energies of protons and oxygen vacancies, defined as the difference between the formation energies in free and bound states; *p_b_* = 1 − (1 − *c*_R_)^2^ and *p_f_* = (1 − *c*_R_)^2^ are the probabilities that an oxygen site is located near acceptor impurities and away from them, respectively.

**Figure 1:**
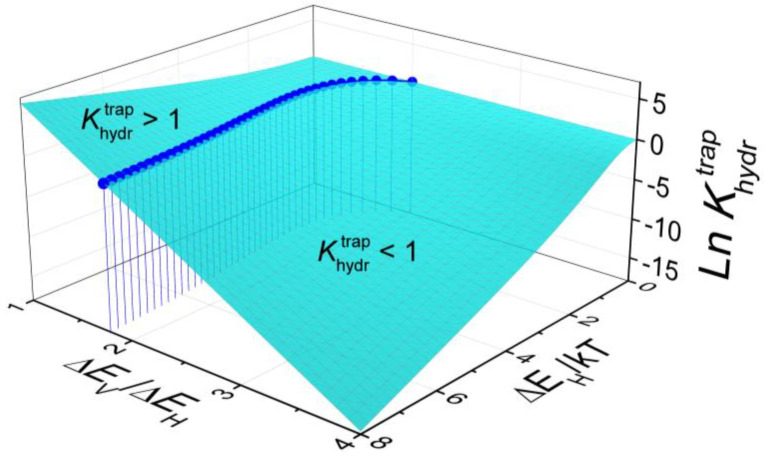
Trapping-related component of the equilibrium constant of hydration as a function of the proton trapping energy Δ*E*_H_/*kT* and the ratio Δ*E*_V_/Δ*E*_H_ for an acceptor-doped perovskite AB_0.9_R_0.1_O_3−δ_. The points on the surface indicate the boundary separating the regions, in which hydration is enhanced ($K_{\mathrm{hydr}}^{\mathrm{trap}}$ > 1) or suppressed ($K_{\mathrm{hydr}}^{\mathrm{trap}}$ < 1) by trapping.

Figure 1 shows the dependence of $K_{\mathrm{hydr}}^{\mathrm{trap}}$ on the proton trapping energy Δ*E* and the ratio Δ*E*_V_/Δ*E*_H_. There are two regions of the Δ*E*_H_ and Δ*E*_V_ values, in which hydration is enhanced ($K_{\mathrm{hydr}}^{\mathrm{trap}}>1$) or suppressed ($K_{\mathrm{hydr}}^{\mathrm{trap}}<1$) by trapping. The boundary between these two regions is determined by $K_{\mathrm{hydr}}^{\mathrm{trap}}=1$. A detailed thermodynamic analysis of hydrogen dissolution taking into account acceptor-bound states of ionic defects is given in Reference [16].

### 2.2. Proton Transport

The consideration of proton transport in this study relies on our recently developed analytical description of proton-hopping conduction in proton-conducting oxides [22]. Let us outline the physical model and the main assumptions underlying this approach.

**Figure 2:**
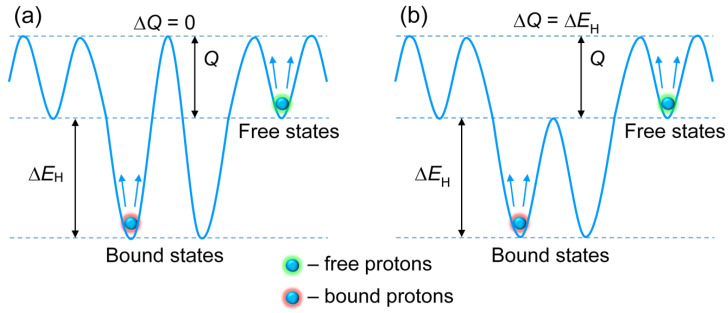
Schematic representation of the two models of the potential energy landscape for proton hopping corresponding to (**a**) Δ*Q* = 0 and (**b**) Δ*Q* = Δ*E*_H_.

The proton migration at elevated temperatures in the studied cubic perovskites AB_1–x_R_x_O_3–δ_ is considered to be the result of thermally activated hopping between neighboring oxygen sites [19,22]. To analyze the effect of acceptor impurities on conduction, we consider two models of the potential energy landscape for proton hopping [22]. The first model implies that acceptor impurities deepen potential wells for protons at the nearest oxygen sites (bound states), but have little effect on the saddle point energies for proton inter-site transitions (Figure 2a). In the second model, impurities considerably reduce both the proton energy at the nearest oxygen sites and the saddle point energy for transitions between neighboring bound sites (Figure 2b).

Under equilibrium, the rate of the thermally activated transition from the occupied site *i* to the empty site *j* is

(4) $$W_{ij}^{0}=\nu_{i}exp\left(-\frac{Q_{ij}}{kT}\right),$$

where the superscript “0” denotes the equilibrium value; *Q_ij_* is the potential barrier for the jump *i* → *j* and ν*_i_* is the prefactor (with dimension of frequency), which is assumed to be the same for all sites: ν*_b_* = ν*_f_* = ν. The barriers *Q_ij_* for the different types of sites in the pairs (*i*, *j*) are: *Q_ff_* = *Q_fb_* = *Q*, *Q_bf_* = *Q* + Δ*E*_H_ and *Q_bb_* = *Q* + Δ*E*_H_ − Δ*Q*. For the potential energy landscapes depicted in Figure 2a,b, the parameter Δ*Q* takes the values of 0 and Δ*E*_H_, respectively.

The energy landscape with Δ*Q* = 0, corresponding to the known lattice gas model with site-energy disorder, was previously used in computer simulations and interpretation of the experimental data on proton dynamics in proton-conducting oxides (see, e.g., Refs. [5,19,20]). The model with Δ*Q* > 0 was also exploited in several works: in Monte Carlo simulations of proton transport and the interpretation of nuclear magnetic resonance data [9,19].

Proton hopping in our work [22] is considered to be governed by the standard master equation, which, in the mean field approximation, gives the rate equation for the occupation probability *f_i_* of site *i*. Under an external electric field, both the rate *W_ij_* of the transitions *i* → *j* and the occupation probability *f_i_* deviate from their equilibrium values $W_{ij}^{0}$ and $f_{i}^{0}$, resulting in a current of proton charge carriers. The calculation of this current is a complex problem due to the effects of disorder and different types of inter-site transitions.

Our approach [22] to the analysis of proton conduction is based on the mapping of the hopping problem onto the random resistor network of Miller and Abrahams [30], and treating it using effective medium theory (see, e.g., Ref. [31]). The local conductances *g_ij_* between pairs of neighboring oxygen sites (*i*, *j* = *f*, *b*) corresponding to our problem can be written as follows [22]:

(5) $$g_{ff}=g_{fb}=g_{bf}=\frac{e^{2}}{kT}\nu f_{f}^{0}exp\left(-\frac{Q}{kT}\right),$$

(6) $$g_{bb}=\frac{e^{2}}{kT}\nu f_{b}^{0}exp\left(-\frac{Q+\Delta E_{H}-\Delta Q}{kT}\right),$$

where $f_{f}^{0}$ and $f_{b}^{0}$ are the equilibrium occupation probabilities for free and bound sites. To obtain the expressions for the conductances (5) and (6), we exploited the detailed balance condition and Boltzmann statistics ($f_{i}^{0}\ll 1$) for protons. The latter is possible because, for the considered moderate dopant content, we can neglect the prohibition for several protons to occupy the same oxygen sites simultaneously [19,22].

In the effective medium approximation, the effective conductance *g*^eff^ of the network of randomly distributed resistors is determined by the equation [31]

(7) $$\int^{\;}\frac{w\left(g\right)\left(g^{\mathrm{eff}}-g\right)}{g+\left(z/2-1\right)g^{\mathrm{eff}}}dg=0,$$

where *w*(*g*) is the probability distribution function for *g_ij_* values; *z* is the coordination number for the network sites (*z* = 8 in our case).

Within the considered model with bound and free sites,

(8) $$w\left(g\right)=\underset{lm}{\sum }p_{lm}\delta \left(g-g_{lm}\right),$$

where *p_lm_* is the probability that two nearest neighboring oxygen sites are of types *l* and *m* (*l*, *m* = *b*, *f*).

For the adopted uniform distribution of acceptor impurities, Equations (5)–(8) have an exact analytical solution for *g*^eff^. The corresponding expression for the macroscopic conductivity is [22]

(9) $$\sigma_{H}=\frac{e}{V_{0}}c_{H}\frac{M_{\mathrm{trap}}}{p_{f}+p_{b}exp\left(\Delta E_{H}/kT\right)}u_{H}^{0}=\frac{e}{V_{0}}c_{H}u_{H}.$$

Here, *V*_0_ is the volume of the formula unit; *u*_H_ is the proton mobility and $u_{H}^{0}$ is the proton mobility in the absence of the interaction between protons and acceptor impurities:

(10) $$u_{H}^{0}=\frac{A_{u}}{T}exp\left(-\frac{Q}{kT}\right),$$

where *A_u_* is the prefactor. The component of the proton mobility associated with the interaction of ionic defects with acceptor impurities is defined as $u_{H}^{\mathrm{trap}}=u_{H}/u_{H}^{0}$.

The function *M*_trap_(*c*_R_, Δ*Q*/*kT*) in Equation (9) is

(11) $$\begin{array}{rl} M_{\mathrm{trap}}=\frac{1}{6}[(4p_{bb} & -1)\exp(\Delta Q/kT)+3-4p_{bb}\\ & +\sqrt{{\left[\left(4p_{bb}-1\right)\exp(\Delta Q/kT)+3-4p_{bb}\right]}^{2}+12\exp(\Delta Q/kT)}], \end{array}$$

where $p_{bb}=c_{R}\left(1+c_{R}-c_{R}^{2}\right)$ is the probability that two nearest neighboring oxygen sites are of type b. The above expression for σ_H_ corresponds to the diagonal component of the conductivity tensor in a crystal with cubic symmetry. In Equation (9) and below, the tensor indices are omitted.

Consider the main features of the proton mobility behavior as a function of dopant content *c*_R_ for the adopted models of the potential energy landscape. The results for the second type of the landscape are given for Δ*Q* = Δ*E*_H_; however, the behavior of *u*_H_(*c*_R_, Δ*Q/kT,* Δ*E*_H_/*kT*) is quite general [22].

**Figure 3:**
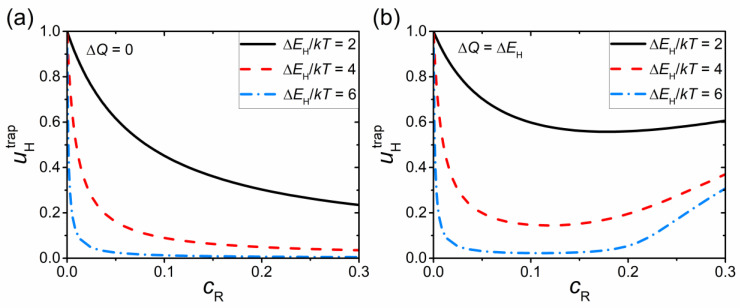
Component of the proton mobility $u_{H}^{\mathrm{trap}}$ related to the effect of acceptor impurities as a function of dopant content *c*_R_ for an acceptor-doped perovskite AB_1__−__x_R_x_O_3__−__δ_. The results are presented for the two models of the potential energy landscape with (**a**) Δ*Q* = 0 and (**b**) Δ*Q* = Δ*E*_H_ for different Δ*E*_H_/*kT* ratios.

At Δ*Q* = 0, *M*_trap_ = 1 and the expression for the proton mobility simplifies: $u_{H}=u_{H}^{0}{\left[p_{f}+p_{b}exp\left(\Delta E_{H}/kT\right)\right]}^{-1}$. In this case, *u*_H_ decreases with increasing the concentration of acceptors *c*_R_ and the proton trapping energy Δ*E*_H_ (see Figure 3a).

In the model of the potential landscape with Δ*Q* = Δ*E*_H_, the barriers for the transitions *b* → *b* are significantly lower than in the first model: *Q_bb_* = *Q_ff_* (see Figure 2). Low barriers *Q_bb_* result in a non-monotonic dependence *u*_H_(*c*_R_), as seen in Figure 3b. At low dopant concentrations, when the clusters of bound sites are isolated, *u*_H_ decreases with increasing *c*_R_, as in the first model. Further increase in *c*_R_ results in the overlapping of the regions of bound states and the formation of an infinite cluster at $c_{R}=c_{R}^{*}$, where $c_{R}^{*}$ is the percolation threshold. At $c_{R}>c_{R}^{*}$, the mobility *u*_H_ increases with increasing *c*_R_ due to a growing contribution of proton transfer over the network of pair-connected bound sites. The $c_{R}^{*}$ value in the considered problem can be found analytically ($\;c_{R}^{*}$ ≈ 0.21). For a more detailed discussion concerning the behavior of *u*_H_, see [22].

Note that the behavior of *u*_H_, predicted within our analytical approach, agrees with the results of Monte Carlo simulations for similar potential energy landscapes [19,20,21]. It should also be noted that, in our consideration, we neglect the correlation effects caused by the interactions between charge carriers. These effects can be significant at high dopant content. However, as the Monte Carlo results showed [19,20], the influence of proton–proton and proton–vacancy interactions on proton conduction is not too significant and can be neglected in many cases up to concentrations *c*_R_~0.20–0.25.

**Figure 4:**
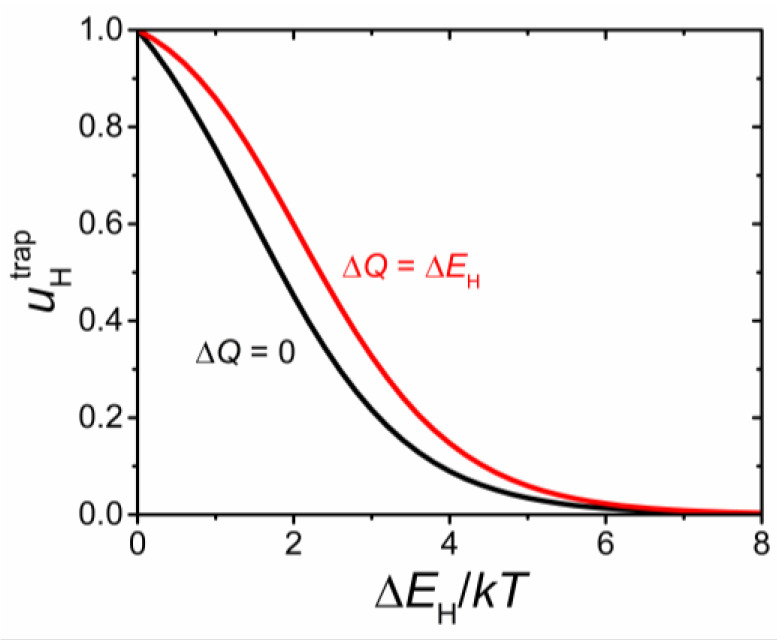
Component of the proton mobility $u_{H}^{\mathrm{trap}}$ related to the effect of acceptor impurities as a function of the proton trapping energy Δ*E*_H_ normalized to *kT* for an acceptor-doped perovskite AB_0.9_R_0.1_O_3__−__δ_. The results are presented for the two models of the potential energy landscape with Δ*Q* = 0 (black line) and Δ*Q* = Δ*E*_H_ (red line).

At moderate dopant content and reasonable values of the parameter Δ*Q* and trapping energies, the difference in the proton mobilities, corresponding to the considered models of the potential energy landscape, is not too significant for most problems [22]. This difference is illustrated in Figure 4 for a perovskite AB_0.9_R_0.1_O_3–δ_. Since altering the heights of the barriers for transitions between bound states has little effect on the results at the considered dopant content and relevant model parameters, further analysis is given for the potential energy landscape with Δ*Q* = 0.

## 3. Results and Discussion

### 3.1. Model Parameters for Barium Stannate

To determine the hydration and proton transfer parameters, which are independent of the interaction between defects and impurities, we used the experimental data on hydrogen dissolution and bulk conductivity for BaSn_0.875_Sc_0.125_O_3–δ_ [7]. The trapping energies of protons Δ*E*_H_ and oxygen vacancies Δ*E*_V_ required for the calculation of hydration and conductivity are taken from the DFT study [28] (see Table 1). Note that we use the same set of trapping energies as in our previous work on the hydration of acceptor-doped BaSnO_3_ [8].

The trapping-independent components of the hydration enthalpy $\Delta H_{\mathrm{hydr}}^{0}$ and entropy $\Delta S_{\mathrm{hydr}}^{0}$ (Equation (2)) obtained by the fitting of the experimental hydration isobars for BaSn_0.875_Sc_0.125_O_3–δ_ are presented in Table 1. The validation of the model by comparison of the theoretical predictions, obtained using the determined parameters, with the hydration data for Y-, In- and Gd-doped BaSnO_3_ is given in our previous study [8]. In general, the exploited theory provides good agreement with the experimental isobars for BaSnO_3_ doped with various acceptors [8]. It is noteworthy that, according to the thermogravimetry measurements [7], the effective and nominal dopant content of the considered oxides differs. The possible reasons for such difference are discussed in more detail elsewhere [14,16]. Henceforth, we use the effective dopant content [7] for the calculation of the hydration and transport properties of barium stannate with nominal composition BaSn_0.875_Sc_0.125_O_3–δ_ (see Table 1).

The activation energy *Q* and prefactor *A*_u_ of the proton mobility in the absence of the interaction with acceptor impurities (Equation (10)) were determined by fitting of the conductivity data for Sc-doped BaSnO_3_ [7]. Since protons provide the dominant contribution to charge transfer at low temperatures [7], only the bulk conductivity data below ~700 K were used for fitting. The parameters *Q* and *A*_u_ were determined for the potential energy landscape for proton hopping with Δ*Q* = 0. As mentioned in Section 2.2, two models of the potential landscape (Figure 2) yield similar results at the moderate dopant content and energy parameters considered in this study. Therefore, here and below, we consider only one model.

### 3.2. Dependence of the Proton Conductivity on the Trapping Energies of Ionic Defects

**Figure 5:**
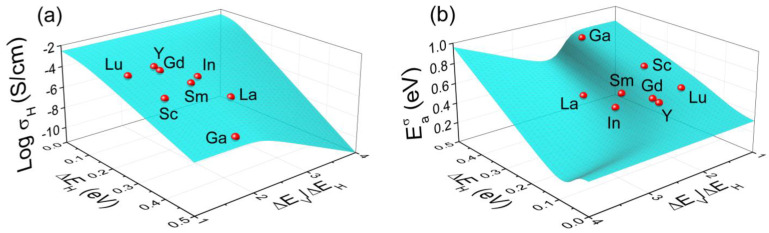
Proton conductivity σ_H_ (**a**) and its activation energy $E_{a}^{\sigma }$ (**b**) as a function of the proton trapping energy Δ*E*_H_ and the ratio Δ*E*_V_/Δ*E*_H_ for BaSn_0.9_R_0.1_O_3__−δ_ in a humidified atmosphere (*p*_H_2_O_ = 0.021 atm, *T* = 500 K). The points on the surfaces correspond to the conductivities and activation energies calculated using the trapping energies for BaSnO_3_ doped with different impurities (see Table 1).

The dependence of the proton conductivity and its activation energy on the trapping energies of protons and oxygen vacancies is shown in Figure 5. The effective activation energy is calculated as

(12) $$E_{a}^{\sigma }=kT^{2}\frac{dln\left(\sigma_{H}T\right)}{dT}.$$

In contrast to hydration, which can be enhanced or suppressed by trapping depending on the relationship between the energies Δ*E*_H_ and Δ*E*_V_ (Figure 1), the proton conductivity σ_H_ is always reduced by the trapping effect at dopant concentrations below the percolation threshold, as illustrated in Figure 5a. At low temperatures, when the oxide is fully hydrated, an increase in the proton trapping energy Δ*E*_H_ results in the reduction of σ_H_ due to a decrease in the proton mobility. However, at higher temperatures, when the oxide is partially hydrated, the dependence σ_H_(Δ*E*_H_) can be non-monotonic (for more details, see [22]). Increasing the trapping energy of oxygen vacancies Δ*E*_V_ shifts the hydration isobars to the low-temperature region [16]. Correspondingly, at a certain Δ*E*_V_ value, when the contribution of oxygen vacancies to the charge neutrality condition becomes noticeable, σ_H_ begins to decrease with increasing Δ*E*_V_, as can be seen in Figure 5a. Such behavior of the proton conductivity results in a significant change in the effective activation energy (Figure 5b). The points on the surfaces in Figure 5 indicate the theoretical values of σ_H_ and $E_{a}^{\sigma }$ calculated using the trapping energies corresponding to particular acceptor impurities.

**Figure 6:**
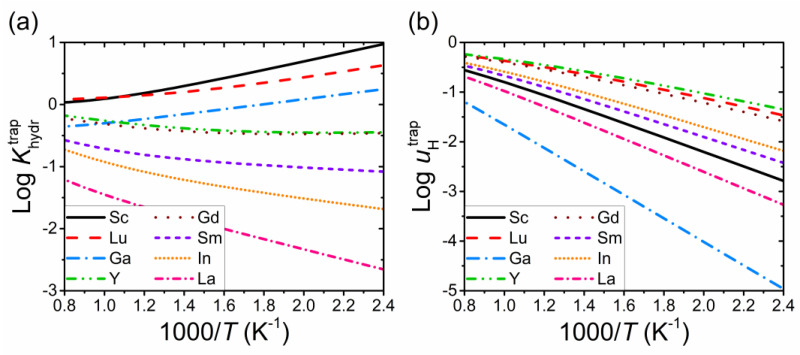
Components of the (**a**) equilibrium constant $K_{\mathrm{hydr}}^{\mathrm{trap}}$ and (**b**) proton mobility $u_{H}^{\mathrm{trap}}$ related to the effect of acceptor impurities as a function of temperature for BaSn_0.9_R_0.1_O_3−δ_. The calculations for each acceptor impurity were performed using the corresponding trapping energies (see Table 1).

The effect of specific dopants on the temperature dependence of the components of the equilibrium constant $K_{\mathrm{hydr}}^{\mathrm{trap}}$ and proton mobility $u_{H}^{\mathrm{trap}}$ related to the interaction of defects with acceptor impurities is illustrated in Figure 6. $K_{\mathrm{hydr}}^{\mathrm{trap}}$ depends on the trapping energies of both protons Δ*E*_H_ and oxygen vacancies Δ*E*_V_. The hydration properties of an oxide can be improved by choosing an acceptor dopant with maximum and minimum values of Δ*E*_H_ and Δ*E*_V_, respectively. Figure 6a shows that maximum equilibrium constant is expected for Sc and Lu, while La provides the worst hydration among the considered dopants. In contrast to $K_{\mathrm{hydr}}^{\mathrm{trap}}$, the proton mobility under the considered conditions is determined only by the trapping of protons and decreases with increasing Δ*E*_H_ (Figure 6b). Accordingly, the highest proton mobility is expected for dopants with the lowest values of Δ*E*_H_. Note that for dopants with large ionic radii, there may be an additional effect of trapping on proton conduction; this will be discussed further in Section 3.5.

In the low-temperature region, when the oxide is fully hydrated, the change in the proton conductivity σ_H_ upon replacement of an acceptor impurity is predominantly determined by the change in the Δ*E*_H_ value. At higher temperatures, when the contribution of oxygen vacancies to the charge neutrality condition is significant, σ_H_ depends on both energies Δ*E*_H_ and Δ*E*_V_. Therefore, the order in which the conductivity value of an oxide doped with different impurities changes can differ at high and low temperatures.

### 3.3. Proton Conductivity as a Function of the Ionic Radius of the Acceptor Dopant

To further elucidate the effect of acceptor impurities on the transport properties of barium stannate, we consider the dependence of the proton conductivity σ_H_ and its effective activation energy $E_{a}^{\sigma }$ on the ionic radius *r*_A_ of the dopant. In our model, this dependence results from the correlation between the radius *r*_A_ and the trapping energies Δ*E*_H_ and Δ*E*_V_. For barium stannate, such correlation was established by the DFT simulation [28].

**Figure 7:**
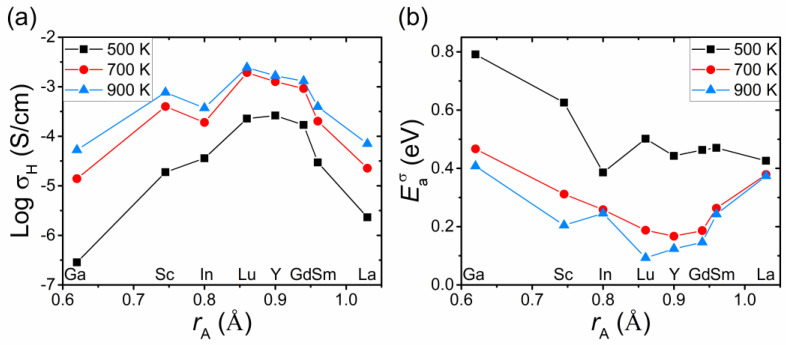
Proton conductivity σ_H_ (**a**) and its effective activation energy $E_{a}^{\sigma }$ (**b**) of BaSn_0.9_R_0.1_O_3−δ_ as a function of the ionic radius *r*_A_ of the dopant (*p*_H_2_O_ = 0.021 atm). The values of σ_H_ and $E_{a}^{\sigma }$ were calculated using the trapping energies for the acceptor dopants from the DFT study [28].

Figure 7 shows the results of the calculations of σ_H_ and $E_{a}^{\sigma }$ for BaSn_0.9_R_0.1_O_3–δ_ with different dopants. As can be seen, the conductivity increases with increasing ionic radius for small dopants (with In being an outlier at elevated temperatures) and decreases with increasing *r*_A_ for large dopants (Figure 7a). The calculated activation energies alter substantially upon varying *r*_A_, and the dependence $E_{a}^{\sigma }$(*r*_A_) is also non-monotonic (Figure 7b).

### 3.4. Effect of Temperature on the Activation Energies and Prefactors of Proton Conductivity and Mobility

**Figure 8:**
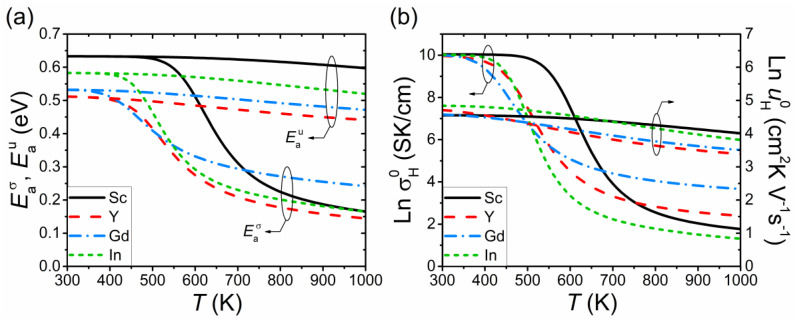
Temperature dependence of the (**a**) activation energy and (**b**) prefactor of the proton conductivity ($E_{a}^{\sigma }$ and $\sigma_{H}^{0}$) and mobility ($E_{a}^{u}$ and $u_{H}^{0}$) in BaSn_0.875_R_0.125_O_3−δ_ (R = Sc, Y, Gd, In) in a humidified atmosphere (*p*_H_2_O_ = 0.021 atm). The calculations were performed for the proton concentrations derived from the thermogravimetry data [7]. The left and right axes in (**b**) show the prefactors of the conductivity ($\sigma_{H}^{0}$) and mobility ($u_{H}^{0}$), respectively.

Figure 8 reports the temperature dependence of the effective activation energy $E_{a}^{\sigma }$ and prefactor $\sigma_{H}^{0}$ of the proton conductivity for Sc-, Y-, Gd- and In-doped BaSnO_3_. The prefactor is determined by

(13) $$ln\left(\sigma_{H}^{0}\right)=\frac{d\left(Tln\left(\sigma_{H}T\right)\right)}{dT}$$

and the expression for $E_{a}^{\sigma }$ is given by Equation (12).

Both parameters $E_{a}^{\sigma }$ and $\sigma_{H}^{0}$ increase with decreasing temperature and attain a saturation limit in the region of complete oxide hydration (*c*_H_ ≈ *c*_R_). Such behavior of $E_{a}^{\sigma }$(*T*) and $\sigma_{H}^{0}$(*T*) is mainly related to the variation in the proton concentration *c*_H_ with temperature. To illustrate this relation, we calculated the temperature dependence of the activation energy $E_{a}^{u}$ and prefactor $u_{H}^{0}$ of the proton mobility using equations similar to (12) and (13). As seen in Figure 8, $E_{a}^{u}$ and $u_{H}^{0}$ weakly depend on *T*, in contrast to a pronounced decline in $E_{a}^{\sigma }$ and $\sigma_{H}^{0}$ with increasing temperature.

As temperature decreases and *c*_H_ approaches the saturation value *c*_R_, $E_{a}^{\sigma }$ (12) and $\sigma_{H}^{0}$ (13), in the region of moderate dopant concentrations, tend to the limits

(14) $$E_{a}^{\sigma ,\;\mathrm{low}\;T}=Q+\Delta E_{H},$$

(15) $$\sigma_{H}^{0,\;\mathrm{low}\;T}=\frac{e}{V_{0}}\frac{c_{R}}{p_{b}}A_{u}M_{\mathrm{trap}}^{0},$$

where $M_{\mathrm{trap}}^{0}$ is the low-temperature limit of the function *M*_trap_ (11). $M_{\mathrm{trap}}^{0}$ = 1 and $M_{\mathrm{trap}}^{0}$ = (1 − 4*p_bb_*)^−1^ for the potential energy landscapes with Δ*Q* = 0 and Δ*Q* = Δ*E*_H_, respectively.

At high temperatures, when the proton concentration is low (*c*_H_ << *c*_R_), the activation energy can be approximated by

(16) $$E_{a}^{\sigma ,\mathrm{high}\;T}=Q+0.5\Delta H_{\mathrm{hydr}}^{0}+\frac{0.5p_{b}\Delta E_{V}}{p_{b}+p_{f}exp\left(-\Delta E_{V}/kT\right)}+kT^{2}\frac{dln\left(M_{\mathrm{trap}}\right)}{dT}.$$

The last term in Equation (16) equals zero for Δ*Q* = 0 and attains a constant value, depending on Δ*E*_H_ and *c*_R_, at high temperatures for Δ*Q* = Δ*E*_H_. Thus, the observed weak temperature dependence of $E_{a}^{\sigma }$ at high *T* in Figure 8a is determined by the third term in Equation (16). In the case of negligible trapping, the high-temperature limit of $E_{a}^{\sigma }$ is constant and equals $Q+0.5\Delta H_{\mathrm{hydr}}^{0}$.

Equations (14) and (15) show that the low-temperature limit of $E_{a}^{\sigma }$ linearly depends on the proton trapping energy Δ*E*_H_ (Figure 8a), while the saturation limit of $\sigma_{H}^{0}$ is the same for all dopants (Figure 8b). As a result, at low temperatures, the activation energy decreases in the order Sc > In > Gd > Y, in accordance with the Δ*E*_H_ values (see Table 1). However, at higher temperatures, the trend is different since the activation energy and prefactor depend on both energies Δ*E*_H_ and Δ*E*_V_. It should be noted that, outside the region of small dopant concentrations, varying *c*_R_ within reasonable limits has virtually no effect on the calculated values of $E_{a}^{\sigma }$ and $\sigma_{H}^{0}$.

Figure 8a demonstrates that in order to obtain the low-temperature limit of the activation energy of the proton conductivity from the experimental dependence σ_H_(*T*), the temperature range should be chosen in the region of complete oxide hydration. However, this can be complicated providing that the oxide hydration is poor and/or the conductivity measurements are performed at elevated temperatures, when *c*_H_ < *c*_R_.

It is important to note that the activation energy is usually determined within the temperature range that exceeds the region of complete oxide hydration. In this case, the obtained temperature-averaged $E_{a}^{\sigma }$ value would be lower than the low-temperature limit determined by Equation (14). For example, the low-temperature limit of the activation energy for Sc-doped BaSnO_3_ equals *Q* + $\Delta E_{H}^{\mathrm{Sc}}$ ≈ 0.63 eV (see Figure 8a). At the same time, the theoretical value of $E_{a}^{\sigma }$ averaged over the temperature range 500–700 K is approximately 0.48 eV, which is close to the result of Wang et al. [7].

### 3.5. Comparison with Experimental Data

**Figure 9:**
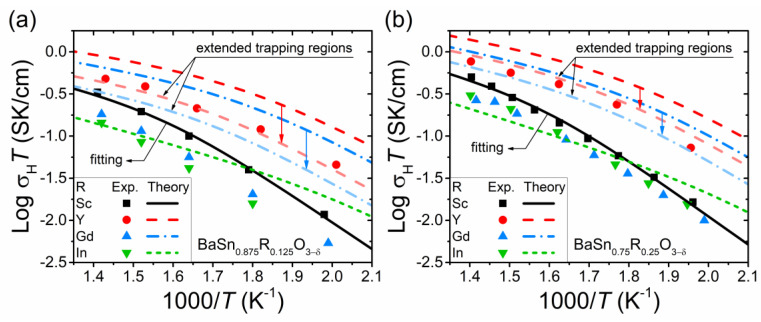
Temperature dependence of the proton conductivity of (**a**) BaSn_0.875_R_0.125_O_3−δ_ and (**b**) BaSn_0.75_R_0.25_O_3−δ_ in a humidified atmosphere (*p*_H_2_O_ = 0.021 atm). The symbols correspond to the experimental data on bulk conductivity in wet Ar [7,29]. The parameters *A*_u_ and *Q* for the proton mobility were determined by fitting of the conductivity data for Sc-doped BaSnO_3_ (Table 1). Solid black lines are the fitting curves. Red, blue and green lines are the theoretical conductivities calculated using the determined parameters and DFT results for the trapping energies [28]. Dopant concentrations *c*_R_ are taken to be equal to the (**a**) effective [7] and (**b**) nominal, *c*_R_ = 0.25, values (since the effective values for BaSn_0.75_R_0.25_O_3−δ_ are unknown). Red and blue arrows indicate the results of the estimates for the oxide with large dopants (Y, Gd) made using the trapping regions around acceptor impurities extended up to the second neighbors.

Figure 9 shows the proton conductivity of Sc-, Y-, Gd- and In-doped BaSnO_3_ calculated within our model, along with the experimental data [7,29]. The conductivity of Sc-doped BaSnO_3_ is a result of the fitting procedure (see Section 3.1). The conductivity of BaSnO_3_ doped with other acceptor impurities is obtained without any fitting, using the determined model parameters (Table 1) and the trapping energies from the DFT study [28]. The calculations for BaSn_0.875_R_0.125_O_3–δ_ were performed using the effective values $c_{R}^{\mathrm{eff}}$ of the dopant content [7]. For BaSn_0.75_R_0.25_O_3–δ_, we used the nominal value *c*_R_ = 0.25 since there are no hydration data for these samples in [29]. 

It should be noted that the conductivities of BaSn_0.875_R_0_._125_O_3–δ_ and BaSn_0.75_R_0.25_O_3–δ_ do not differ significantly under the considered conditions. Such a weak dependence σ_H_(*c*_R_) outside the regions of small and large *c*_R_ values is not unusual; it was experimentally observed for other proton-conducting perovskites (see, e.g., Ref. [32]). The Monte Carlo simulations also showed that, under certain conditions, the dependence σ_H_(*c*_R_) can be relatively weak at intermediate dopant concentrations [19,20]. This effect can be explained by the mutually compensating influence on the conductivity of two factors—an increase in the proton concentration *c*_H_ and a decrease in the proton mobility *u*_H_ with increasing *c*_R_. Such a decreasing dependence *u*_H_(*c*_R_) at the considered moderate dopant content is caused by the trapping effect (see Figure 3 and Refs. [19,20]).

It can be seen from Figure 9 that the shifts in the calculated proton conductivity for In and Y relative to Sc generally follow the experimental data, although the slopes of the conductivity curves for In are somewhat different in the low-temperature region. The agreement between theory and experiment for Gd-doped BaSnO_3_ is worse than for the oxide with other dopants. The reasons for this discrepancy in the case of Y and Gd can be partially explained by their large ionic radius, as will be seen further below. Another important factor that can lead to lower conductivity values, as compared to the theoretical results, is slow kinetics caused by the high density of the samples. In particular, it can hinder the attainment of the theoretically expected degree of hydration, especially at low temperatures. For example, the relative density of BaSn_0.875_R_0.125_O_3–δ_ (R = In, Gd) [7] and BaSn_0.75_In_0.25_O_3–δ_ [29] was above 98%.

According to a number of DFT studies for BaSnO_3_ [13,28], in the case of acceptor dopants with large ionic radii, the trapping energies of protons and oxygen vacancies in the first and second neighbor positions can be comparable. In order to roughly estimate the implications of this effect, we extend the trapping regions around such impurities up to the second neighbors, accordingly redefining the probabilities *p_f_* and *p_b_*. The total number of proton positions in such trapping regions is large, and we are beyond the applicability of our theory (especially if the effect of impurities on the saddle point energies for inter-site transitions is significant, as in the potential landscape model with Δ*Q* = Δ*E*_H_). Nevertheless, to demonstrate a possible trend, we provide these estimates for the potential landscape with Δ*Q* = 0. As shown in Figure 9, the extension of the trapping region leads to good agreement between theory and experimental data for Y-doped BaSnO_3_. However, the results for Gd-doped BaSnO_3_ still do not agree quite well with the experiment, although the calculated conductivity values become closer to the experimental data.

In another study, Li and Nino [33] measured the bulk conductivities for BaSn_0.9_R_0.1_O_3–δ_ (R = In, Lu, Er, Y, Gd) under oxidizing and reducing conditions. The results indicate that the order in which the conductivity corresponding to different acceptor impurities changes is quite different from that obtained by Wang et al. [7,29]. However, the external conditions of the conductivity measurements in Reference [33] differed from those in the experiments [7,29], which were carried out in a humidified Ar atmosphere. According to the EMF measurements [33], a significant contribution to the total conductivity in oxidizing and reducing atmospheres is provided by electronic charge carriers. Since this contribution can also differ for samples with different dopants, a comparison of our theoretical results with the bulk conductivity data [33] would be incorrect.

**Figure 10:**
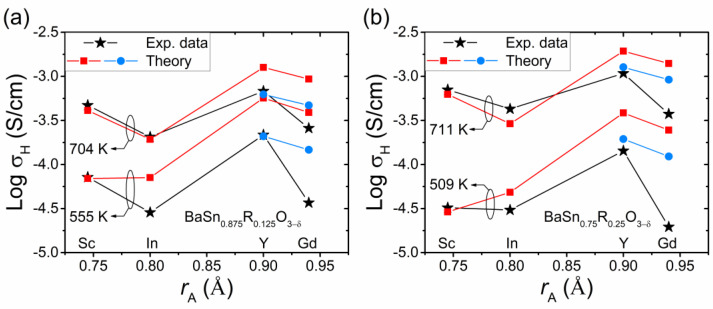
Proton conductivity σ_H_ of (**a**) BaSn_0.875_R_0.125_O_3−δ_ and (**b**) BaSn_0.75_R_0.25_O_3−δ_ as a function of the ionic radius *r*_A_ of the dopant in a humidified atmosphere (*p*_H_2_O_ = 0.021 atm). The black and red symbols are the experimental conductivities [7,29] and the theoretical values calculated within our model, respectively. The blue symbols are the results of the estimates with the trapping regions around impurities extended up to the second neighbor positions. The calculations were performed using the (**a**) effective, $c_{R}^{\mathrm{eff}}$, [7] and (**b**) nominal, *c*_R_ = 0.25, dopant concentrations.

We now turn to the dependence of the proton conductivity on the ionic radius of the dopant *r*_A_. To compare the results with experiments, the bulk conductivity data for Sc-, In-, Y- and Gd-doped BaSnO_3_ [7,29] and the calculated conductivities are plotted as a function of *r*_A_ in Figure 10. It is seen that the behavior of the theoretical conductivities correlates well with the experimental data, including the downward shift for In at elevated temperatures. The estimates of σ_H_(*r*_A_) for the oxide with large dopants Y and Gd were also made using the extended trapping regions around acceptor impurities, see above. The obtained values of σ_H_ (blue points in Figure 10) are shifted downwards and closer to the experimental data.

A non-monotonic dependence σ_H_(*r*_A_), similar to that predicted by our model for acceptor-doped BaSnO_3_ (see Figure 7a and Figure 10), was also experimentally observed for perovskites BaZrO_3_ [10,17] and BaCeO_3_ [34] doped with different acceptor impurities.

## 4. Conclusions

We have applied our recently developed statistical theory of proton-hopping conduction in oxide perovskites to reveal the role of the interaction between ionic defects and acceptor impurities in proton transport in acceptor-doped barium stannate. Accounting for this interaction within the proposed approach allowed us to explain the observed behavior of the bulk proton conductivity σ_H_ of BaSn_1–x_R_x_O_3–δ_. The experimental dependences of σ_H_ on temperature, type of acceptor impurity and its ionic radius are described reasonably well. A number of results concerning the influence of impurities on proton conductivity and mobility are quite general for perovskites with moderate dopant content. For example, in the low-temperature region of complete oxide hydration, the main effect of the interaction between acceptor impurities and ionic defects on the behavior of σ_H_ is due to the proton trapping. In the low-temperature limit, the effective activation energy $E_{a}^{\sigma }$ of σ_H_ increases linearly with increasing the proton trapping energy. At higher temperatures, $E_{a}^{\sigma }$ depends on the trapping energies of both protons and oxygen vacancies and decreases with increasing temperature. The predicted non-monotonic dependence of σ_H_ on the dopant ionic radius is observed not only for BaSnO_3_, but also for other acceptor-doped perovskites. Our findings contribute to the understanding of the role of acceptor impurities in proton transport in oxides and can be useful for selecting optimal acceptor doping for proton-conducting materials.

## Figures and Tables

**Table 1:** Model parameters for R-doped BaSnO_3_.

Trapping energies of protons and oxygen vacancies for BaSnO_3_ doped with different acceptor impurities [28]	$\Delta E_{H}^{\mathrm{Ga}}$$=0.47\;\mathrm{eV},\;\Delta E_{V}^{\mathrm{Ga}}$ = 0.86 eV
	$\Delta E_{H}^{\mathrm{Sc}}$$=0.29\;\mathrm{eV},\;\Delta E_{V}^{\mathrm{Sc}}$ = 0.44 eV
	$\Delta E_{H}^{\mathrm{In}}$$=0.24\;\mathrm{eV},\;\Delta E_{V}^{\mathrm{In}}$ = 0.56 eV
	$\Delta E_{H}^{\mathrm{Lu}}$$=0.18\;\mathrm{eV},\;\Delta E_{V}^{\mathrm{Lu}}$ = 0.25 eV
	$\Delta E_{H}^{Y}$$=0.17\;\mathrm{eV},\;\Delta E_{V}^{Y}$ = 0.32 eV
	$\Delta E_{H}^{\mathrm{Gd}}$$=0.19\;\mathrm{eV},\;\Delta E_{V}^{\mathrm{Gd}}$ = 0.36 eV
	$\Delta E_{H}^{\mathrm{Sm}}$$=0.26\;\mathrm{eV},\;\Delta E_{V}^{\mathrm{Sm}}$ = 0.55 eV
	$\Delta E_{H}^{\mathrm{La}}$$=0.33\;\mathrm{eV},\;\Delta E_{V}^{\mathrm{La}}$ = 0.82 eV
Effective dopant content for barium stannate with nominal composition BaSn_0.875_R_0.125_O_3–δ_ [7]	$c_{\mathrm{Sc}}^{\mathrm{eff}}$$=0.1046,\;c_{Y}^{\mathrm{eff}}$ = 0.0836
	$c_{\mathrm{Gd}}^{\mathrm{eff}}$$=0.0996,\;c_{\mathrm{In}}^{\mathrm{eff}}$ = 0.0769
Enthalpy and entropy of hydration in the absence of trapping	$\Delta H_{\mathrm{hydr}}^{0}$ = –73 kJ mol^−1^
	$\Delta S_{\mathrm{hydr}}^{0}$ = –110 J mol^−1^K^−1^
Activation energy and prefactor of the proton mobility in the absence of the interaction with acceptor impurities	*Q* = 0.34 eV
	*A*_u_ = 19 cm^2^K V^−1^s^−1^

## Data Availability

Not applicable.
